# Differentially expressed proteins in positive versus negative HNSCC lymph nodes

**DOI:** 10.1186/s12920-018-0382-6

**Published:** 2018-08-29

**Authors:** Alessandra Vidotto, Giovana M. Polachini, Marina de Paula-Silva, Sonia M. Oliani, Tiago Henrique, Rossana V. M. López, Patrícia M. Cury, Fabio D. Nunes, José F. Góis-Filho, Marcos B. de Carvalho, Andréia M. Leopoldino, Eloiza H. Tajara

**Affiliations:** 1 Departamento de Biologia Molecular, Faculdade de Medicina (FAMERP), Av. Brigadeiro Faria Lima, 5416, Vila São Pedro, São José do Rio Preto, SP CEP 15090-000 Brazil; 2 Departamento de Biologia, Instituto de Biociências, Letras e Ciências Exatas (IBILCE), Universidade Estadual Paulista (UNESP), R. Cristóvão Colombo, 2265, São José do Rio Preto, SP CEP 15054-000 Brazil; 3 Instituto do Câncer de São Paulo Octavio Frias de Oliveira – ICESP, Av. Dr. Arnaldo, 251 - Cerqueira César, São Paulo, SP CEP 01246-000 Brazil; 4 Faculdade Ceres (Faceres), Av. Anísio Haddad, 6751, São José do Rio Preto, SP CEP 15090-305 Brazil; 5 Departamento de Estomatologia, Faculdade de Odontologia, Universidade de São Paulo, Av. Prof. Lineu Prestes, 2227, São Paulo, SP CEP 05508-000 Brazil; 6 Instituto do Câncer Arnaldo Vieira de Carvalho, R. Dr Cesário Mota Jr, 112, São Paulo, SP CEP 01221-020 Brazil; 7 Departamento de Cirurgia de Cabeça e Pescoço, Hospital Heliópolis, R. Cônego Xavier, 276, São Paulo, SP CEP 04231-030 Brazil; 8 Departamento de Análises Clínicas, Toxicológicas e Bromatológicas, Faculdade de Ciências Farmacêuticas, Universidade de São Paulo, Avenida do Café, s/n, Ribeirão Preto, SP CEP 14040-903 Brazil; 9 Departamento de Genética e Biologia Evolutiva, Instituto de Biociências, Universidade de São Paulo, R. do Matão, 321, São Paulo, SP CEP 05508-090 Brazil

**Keywords:** Head and neck carcinoma, Metastasis, Lymph node, Proteomics

## Abstract

**Background:**

Lymph node metastasis is one of the most important prognostic factors in head and neck squamous cell carcinomas (HNSCCs) and critical for delineating their treatment. However, clinical and histological criteria for the diagnosis of nodal status remain limited. In the present study, we aimed to characterize the proteomic profile of lymph node metastasis from HNSCC patients.

**Methods:**

In the present study, we used one- and two-dimensional electrophoresis and mass spectrometry analysis to characterize the proteomic profile of lymph node metastasis from HNSCC.

**Results:**

Comparison of metastatic and non-metastatic lymph nodes showed 52 differentially expressed proteins associated with neoplastic development and progression. The results reinforced the idea that tumors from different anatomical subsites have dissimilar behaviors, which may be influenced by micro-environmental factor including the lymphatic network. The expression pattern of heat shock proteins and glycolytic enzymes also suggested an effect of the lymph node environment in controlling tumor growth or in metabolic reprogramming of the metastatic cell. Our study, for the first time, provided direct evidence of annexin A1 overexpression in lymph node metastasis of head and neck cancer, adding information that may be useful for diagnosing aggressive disease.

**Conclusions:**

In brief, this study contributed to our understanding of the metastatic phenotype of HNSCC and provided potential targets for diagnostic in this group of carcinomas.

**Electronic supplementary material:**

The online version of this article (10.1186/s12920-018-0382-6) contains supplementary material, which is available to authorized users.

## Background

Metastases are the main cause of death in cancer patients [1]. The power of these malignant cells to kill their hosts resides in their ability to leave the primary tumor, disseminate and invade ectopic sites, as well as to exhibit self-renewal and uncontrollable growth, leading to painful and incurable secondary tumors. In recent years, many data have revealed the determining factors mediating this destructive cascade, which include an extensive and growing list of genetic and epigenetic alterations [2].

In the initial steps of metastatization, tumor cell mutations and signals released by the stromal cells may cooperate to reduce cell-cell adhesion and to promote migration of epithelial neoplastic cells [3]. These events are similar to those of an important reversible differentiation program named epithelial-mesenchymal transition (EMT), which occurs during embryogenesis and have also been implicated in tumor invasion and metastasis [4].

As the tumor grows, low oxygen tension stimulates a proangiogenic response [5]. Due to cytokines secreted by neoplastic and stromal cells, endothelial cells from pre-existing blood vessels synthesize proteases, allowing their migration through the stroma [6]. These migrating endothelial cells proliferate and generate new vessels, which can supply oxygen and nutrients to sustain cancer growth and are an important route for metastasis [7]. Lymphatic vessel formation, a common event in various inflammatory conditions, is also stimulated in some human cancers [8] and evolves to an important route of spread of tumors cells to regional lymph nodes [9].

The lymphatic network is more permissive for metastatic spread than the blood vascular system because (a) the basement membranes of the vessels are incomplete, (b) their capillaries exhibit a single endothelial cell layer not surrounded by pericytes and (c) have intercellular valve-like structures that facilitate the uptake of cells [10]. In addition to the permissive structure, the hydrostatic pressure of the lymphatic system is lower compared to blood circulation, reducing the mechanical challenge [11]. Otherwise, lymph is richer in immune response factors which, although insufficient to destroy tumor cells [12], may play an important role in selecting immune resistance phenotypes [13]. Examples of tumors that frequently metastasize to regional lymph nodes via lymphatic route instead of spreading to distant sites are the head and neck carcinomas (HNSCC) [14], a group of neoplasms nearly always associated with chronic inflammation.

Arrival at a secondary site does not ensure success for most metastatic cells. The processes of extravasation and seeding require specific tumor characteristics and receptive conditions. To increase the chances of a favorable outcome, it has been suggested that target sites are prepared in advance by long-distance interaction with the primary tumor [15, 16]. The pattern of metastatic seeding and colonization is not random and, depending on the primary site, tumor cells spread to particular organ sites more frequently than to others [17].

The mechanisms involved in metastasis organotropism are not completely known but chemokines and their receptors, as well as circulation patterns and structural features of local capillaries should be important contributors for the process [18]. Several critical genes driving organ-specific metastases have been described in different tumors [19, 20]. However, a number of questions remain unanswered. For example, in head and neck carcinomas, regional lymph nodes are the preferential target sites and distant metastases are a late and infrequent finding [21]. Why HNSCCs have this behavior and why small cell carcinomas of the head and neck [22] and several tumors of salivary gland [23], located in the same anatomical site, typically have distant metastases? The answer probably lies in the characteristics of the metastatic cell as well as in its interaction with the microenvironment.

Considering (a) the atypical feature of HNSCC to remain a locoregional disease, (b) the limitations of relapse risk assessment and (c) clinical and histological criteria for the diagnosis of lymph node spread, still the most powerful prognostic factor for these cancers [24], it is urgently necessary to define appropriate biomarkers of the metastatic phenotype for this group of diseases. In the present study, we aimed to characterize the proteomic profile of lymph node metastasis from HNSCC using one- and two-dimensional electrophoresis and mass spectrometry analysis.

## Methods

### Tissue samples

Thirty-two samples of lymph nodes (12 non-metastatic or N0 and 20 metastatic or N+) were obtained from patients with surgically resected head and neck squamous cell carcinomas of three anatomical subsites classified according to the 10th edition of the International Classification of Diseases-10: C02 = other and unspecified parts of tongue; C04 = floor of mouth; C32 = larynx. Ten of the lymph node samples were derived from patients with tongue, 13 from floor of the mouth and 9 from larynx carcinomas and their extracted proteins were analyzed by either one-dimensional gel (1-D) or two-dimensional (2-D) gel electrophoresis. An overlapping set of 22 samples was analyzed by Western blot. Immunohistochemical staining was also performed using formalin-fixed, paraffin-embedded blocks (14 lymph nodes and 9 surgical margins) and tissue microarray slides (65 primary tumors) containing samples from C02, C03 (gum), C04, C32 and C06 (other and unspecified parts of mouth) neoplasms. Therefore, two anatomical sites were analyzed: oral cavity (C02, C03, C04, C06 subsites) and larynx (C032).

Surgical specimens were obtained before radio- or chemotherapy by the Head and Neck Genome Project (GENCAPO), a collaborative consortium of research groups from hospitals and universities in São Paulo State, Brazil, whose aim is to develop clinical, genetic and epidemiological analysis of head and neck squamous cell carcinomas.

Immediately after surgery, part of specimen was frozen in liquid nitrogen and stored at − 80 °C, and part was fixed in formalin for immunohistochemistry or routine histopathological examination. Frozen sections of the lymph nodes were analyzed to confirm the presence (N+) or absence (N0) of tumor cells. The primary tumors were classified by the Tumor-Node-Metastases (TNM) system [25]. A full description of the clinicopathological data is provided in Additional file 1.

The study protocol was approved by each institutional review board and by the National Committee on Ethics in Research/CONEP (reference number 1763/05, 18/05/2005). All patients provided written informed consent.

### Proteomic approaches

#### Sample preparation

Sample preparation was performed according to the protocol described by de Marqui et al. [26], with modifications. In brief, lymph node samples were cut into small pieces and washed with 500 μL of lysis buffer containing 7 M urea, 2 M thiourea, 4% CHAPS detergent, 65 mM DTT, and 0.2% carrier ampholytes. The specimens were disrupted by sonication twice for 1 min at 0 °C and vortexed vigorously for approximately 2 min. The lysates were centrifuged at 10,000 g for 3 min at 4 °C. Protein concentration of the resulting supernatant was determined by the Bradford method [27]. The protein samples were stored in aliquots at − 80 °C.

To optimize the experiments with 32 samples in triplicates, lymph node samples were pooled according to the presence or absence of tumor cells and according to the anatomic subsite, namely tongue, floor of the mouth, and larynx (Additional file 2). The 6 pools (A-F) combined equal amounts of protein from each sample, resulting in 100 μg and 1500 μg per pool for one-dimensional gel electrophoresis (1-DE) and two-dimensional gel electrophoresis (2-DE) gels, respectively.

#### One-dimensional gel electrophoresis (1-DE)

Two protein pools (E and F) of 3 N0 and 6 N+ lymph nodes from larynx carcinomas were analyzed by 1-DE. Under reducing conditions, 100 μg of each protein pool were denatured at 96 °C for 5 min in 5X loading buffer with β-mercaptoethanol and loaded on one-dimensional 12% resolving/5% stacking sodium dodecyl sulfate (SDS) - polyacrylamide gel (PAGE), according to Laemmli [28]. Electrophoresis was carried out on a vertical electrophoresis apparatus (SE 400 Vertical Unit, GE Healthcare, Uppsala, Sweden) at 120 V. Proteins were detected by Coomassie Blue staining, and the molecular mass was estimated using molecular weight standard proteins of 14.4–97 kDa (LMW Calibration Kit for SDS Electrophoresis, GE Healthcare).

#### Two-dimensional gel electrophoresis (2-DE)

Three protein pools of N0 and three protein pools of N+ lymph nodes from patients with tongue (pools A and B), floor of the mouth (pools C and D) or larynx carcinomas (pools E and F) were analyzed by 2-DE, according to de Marqui et al. [26], with modifications. Proteins were precipitated using ice-cold acetone 100%, and centrifuged at 13,000 g for 5 min at 4 °C. Aliquots containing approximately 1500 μg of protein were diluted with rehydration buffer [8 M urea, 2% *w*/*v* CHAPS, 0.6% w/v DTT, 0.5% *v*/v immobilized linear pH gradient (IPG) buffer pH 3–10, trace of bromophenol blue] to a total volume of 250 μL and loaded onto an IPG strip (13 cm, pH 3–10 L, GE Healthcare).

After isoelectric focusing/IEF (total of 26,500 V-hours at 20 °C, 50 mA/strip) on an IPGphor apparatus (GE Healthcare), IPG strips were incubated in the equilibration solution (6 M urea, 50 mM Tris-HCl pH 8.8, 30% v/v glycerol, 2% w/v SDS, trace of bromophenol blue) containing 1% w/v DTT, followed by incubation in the same solution containing 2.5% w/v iodoacetamide instead of DTT. IPG strips were sealed on top of 12.5% SDS-polyacrylamide gel 0.5% w/v low-melting agarose in SDS running buffer.

Electrophoresis was performed in a Hoefer SE 600 Ruby vertical electrophoresis unit (GE Healthcare) at 15 mA/gel for 30 min and 30 mA/gel for 7 h at 10 °C. The samples were run in triplicate and the LMW Calibration Kit was used as a molecular mass standard. After Coomassie blue staining, the gels were scanned using an ImageScanner (GE Healthcare) and the images were analyzed using the ImageMaster 2D Platinum software, version 6.0 (GE Healthcare). Gels from N0 and N+ groups of each anatomical subsite were matched to a reference gel. Spot quantification was based on the spot volume as percentage of the total volume of all spots in the gel. For each anatomical subsite, differential image analysis was carried out by matching spots from gel triplicates of each group (N0 and N+). Differences between groups were evaluated statistically by using the Student’s *t*-test with *p* < 0.05 as significant.

#### In-gel protein digestion and mass spectrometry (MS)

Sequential slices of 1-DE gels and differentially expressed protein spots from 2-DE gels were manually cut out from the gels. The samples were mixed with 50% acetonitrile (ACN)/25 mM ammonium bicarbonate solution and dehydrated with ACN for 15 min. Acetonitrile was discarded and the gel pieces were dried in a vacuum centrifuge for 30 min. Gel pieces were digested with trypsin and incubated for 24 h at 37 °C. Peptides were extracted with 1% trifluoroacetic acid (TFA) for 12 h and 1% TFA/50% ACN for 2 h. The supernatants were mixed and concentrated in a vacuum centrifuge to approximately 5–10 μL.

Digested samples from 1-DE gels were applied to a C18 (100 μm X 100 mm) RP-nanoUPLC (nanoAcquity, Waters, Milford, MA, USA) coupled with a Q-TOF (Quadrupole Ion Trap - Time of Flight) Ultima mass spectrometer (Waters) with nano-electrospray source at a flow rate of 0.6 μL/min. The gradient condition was developed with 0–50% acetonitrile in 0.1% formic acid for 60 min. The instrument was operated in the ‘top three’ mode and the spectra were acquired using the MassLynx software version 4.1 (Waters). The raw data files were processed to peak list with the Mascot Distiller software, version 2.2.1.0 (Matrix Science, London, UK). Mascot search results were exported to Scaffold software (version 3.06, Proteome Software Inc., Portland, OR, USA) for validation. Protein and peptide identification probabilities were set up at > 95%, with one minimum peptide. The samples were grouped in metastatic and non-metastatic, the spectral counts were normalized and proteins with a fold change ≥2.0 were considered to be differentially expressed. Fold change was calculated by Scaffold software (according to [29]).

After trypsin digestion, the peptide samples from 2-DE gels were placed into matrix solution (10 mg/mL α-cyano-4-hydroxycinnamic acid, 0.1% *v*/v TFA in 50% v/v ACN) in a 1:1 (v:v) ratio, spotted on a stainless steel sample plate and analyzed by a MALDI-Q-TOF (Matrix Assisted Laser Desorption Ionization - Quadrupole Ion Trap - Time of Flight) Premier mass spectrometer (Waters). Mascot Daemon (version 2.2.0, Matrix Science) was used to search the NCBI non-redundant database with the parameters: enzyme, trypsin; allowed number of missed cleavages, 1; fixed modification, carbamidomethylation on cysteine; variable modification, oxidation on methionine; peptide tolerance, 0.1 Da; MS/MS tolerance, 0.1 Da; monoisotopic masses.

#### Metabolic pathways, associated ontologies and expression data

The set of genes encoding differentially expressed proteins was imported into HNdb [30], a head and neck database that provides information on genes and proteins involved in head and neck squamous cell carcinoma, covering data on genomics, transcriptomics, proteomics, literature citations and also cross-references of external databases. Using this database, the genes were linked to KEGG [31] metabolic pathways, associated ontologies [32], and microarray data [33].

The set of genes was also functionally clustered using DAVID [34, 35], a database for annotation, visualization and integrated discovery. The one-tail Fisher Exact Probability Value was used for gene-enrichment analysis, and Bonferroni and Benjamini–Hochberg corrected *p*-values less than 0.05 were considered significant.

### Immunodetection

In order to validate the proteomic findings, a literature search was performed to select candidate targets showing an unclear role in head and neck tumorigenesis or involved in the development and progression of head and neck neoplasms but never evaluated in their lymph node metastasis. Using these criteria, two proteins (epidermal-type fatty acid-binding protein or E-FABP, and annexin A1 or ANXA1) were selected to be validated.

#### Western blot

The expression of E-FABP was analyzed by Western blot in a subset of 22 individual samples (11 N0 and 11 N+ lymph nodes from 8 tongue, 8 floor of the mouth and 6 larynx carcinomas). The antibodies used were polyclonal primary anti-E-FABP (ab37267, Abcam, Cambridge, MA, USA) diluted 1:500, and monoclonal primary anti-β-actin antibody (A1978 Sigma-Aldrich, Saint Louis, MO, USA) diluted 1:5000. In brief, protein samples (10 μg) were subjected to SDS-PAGE (12% resolving gel with 5% stacking gel) under denaturing conditions at 120 V for 120 min, using a Mini-Protean 3 Cell Electrophoresis System (BioRad, Hercules, CA, USA). The molecular weight ladder was the PageRuler™ Prestained Protein Ladder (SM0671, Fermentas Life Sciences, Vilnius, Lithuania).

Samples were transferred electrophoretically (90 V for 90 min) to polyvinylidene difluoride (PVDF) membranes (Immobilon-P Membrane, Millipore, Bedford, MA, USA) by using transfer buffer (25 mM Tris, 0.2 M glycine, 20% *v*/v methanol). Antibodies were detected using Western Breeze chromogenic system (Invitrogen, Carlsbad, CA, USA) and the blots were then scanned and analyzed using a Kodak Gel Logic 2200 Digital Imaging System (Carestream Health, Rochester, NY, USA).

#### Immunohistochemistry

Immunohistochemical analysis of a tissue microarray (TMA) with duplicate tissue cores of 65 primary oral squamous cell carcinoma samples was carried out by a polymer-based immunohistochemistry method using rabbit polyclonal antibody anti-E-FABP (ab37267), at a dilution of 1:500. Nine tissue slides containing archival formalin-fixed, paraffin-embedded tissue (FFPE) sections of surgical margins were used to establish a cut off value level for positivity. After deparaffinization and rehydration in xylene and graded ethanol, the slides were immersed in 10 mM citrate buffer (pH 6.0) and heated in a water bath (97 °C, 20 min) for antigen epitope retrieval. Endogenous peroxidase activity was blocked with methanol containing 3% hydrogen peroxide for 30 min. Specimens were incubated overnight with the primary antibody in a humidity-controlled chamber. The sections were washed twice with PBS and Tween 0.25% at room temperature. Immune complexes were subsequently treated using EnVision+Dual Link System-HRP (K4061, DAKO, Fisher Scientific, Hampton, NH, USA), and DAB (3,3′-diaminobenzidine) in chromogen solution (K3468; DAKO, Fisher Scientific). Counterstaining was performed with Mayer’s hematoxylin. Nuclear and cytoplasmatic staining of the epithelial cells was considered specific.

Percentage of positive cells in each TMA spot was scored as follows: 0 or negative (not detectable or detectable in less than 5% of tumor cells), 1 (labeling of more than 5% and less than 10% of tumor cells), 2 (labeling of more than 10% and less than 50% of tumor cells), 3 (labeling of more than 50% and less than 75% of tumor cells), 4 (widely and highly expressed in more than 75% of the tumor cells), at × 400 magnification. The intensity of immunoreaction was scored as negative (0), mild (1), moderate (2) and intense (3). The percentage of positive tumor cells and the staining intensity then were multiplied to produce an E-FABP score for each case. Cases with a final score > 9.4 (the average score from normal tissue) were defined as positive.

Immunohistochemical analysis of 14 lymph node specimens from patients with C02, C04 and C32 tumors was also performed to investigate the expression of annexin A1. Two-micrometer FFPE sections were processed by deparaffinization, rehydration and antigen epitope retrieval, as described above. Endogenous peroxidase and non-specific epitopes were blocked with 3% hydrogen peroxide and 5% bovine serum albumin in phosphate-buffered saline (BSA-PBS) for 30 min, respectively. The slides were then incubated overnight at 4 °C with rabbit polyclonal anti-ANXA1 (71–3400, Thermo Fisher Scientific, Waltham, MA, USA) at a dilution of 1:2000, in 1% BSA. Some sections were incubated with 1% BSA instead of the primary antibody to provide a negative control of the reaction. After washing, sections were incubated with the secondary biotinylated antibody (959943-B, Thermo Fisher Scientific). Positive staining was detected using a peroxidase-conjugated streptavidin complex and the color was developed using DAB substrate (002014, Thermo Fisher Scientific). Finally, sections were counterstained with hematoxylin and mounted. ANXA1 immunostaining was evaluated by densitometric analysis conducted using an Axioskop 2-Mot Plus Microscope and AxioVision 4.8 software (Carl Zeiss, Jena, Germany) on an arbitrary scale from 0 to 255. Data were expressed as mean ± standard error.

## Results

### Casuistic

Of the 32 lymph node samples evaluated by 1-DE/MS, 2-DE/MS, 12 were derived from patients with N0 and 20 with N+ tumors classified as: 10 tongue, 13 floor of the mouth and 9 larynx carcinomas (C02, C04, C32, respectively). The samples were combined in six pools and analyzed using 1-DE and/or 2-DE and mass spectrometry (Additional file 2). The mean age of the patients was 60.1 years (range, 45–79 years), and the male/female sex ratio was 9.7:1. Most patients were smokers or former smokers (28/32) and had a history of chronic alcohol consumption (29/32) (Additional file 1).

### Proteomic approaches

#### One- and two-dimensional gel electrophoresis (1-DE and 2-DE)

**Fig. 1:**
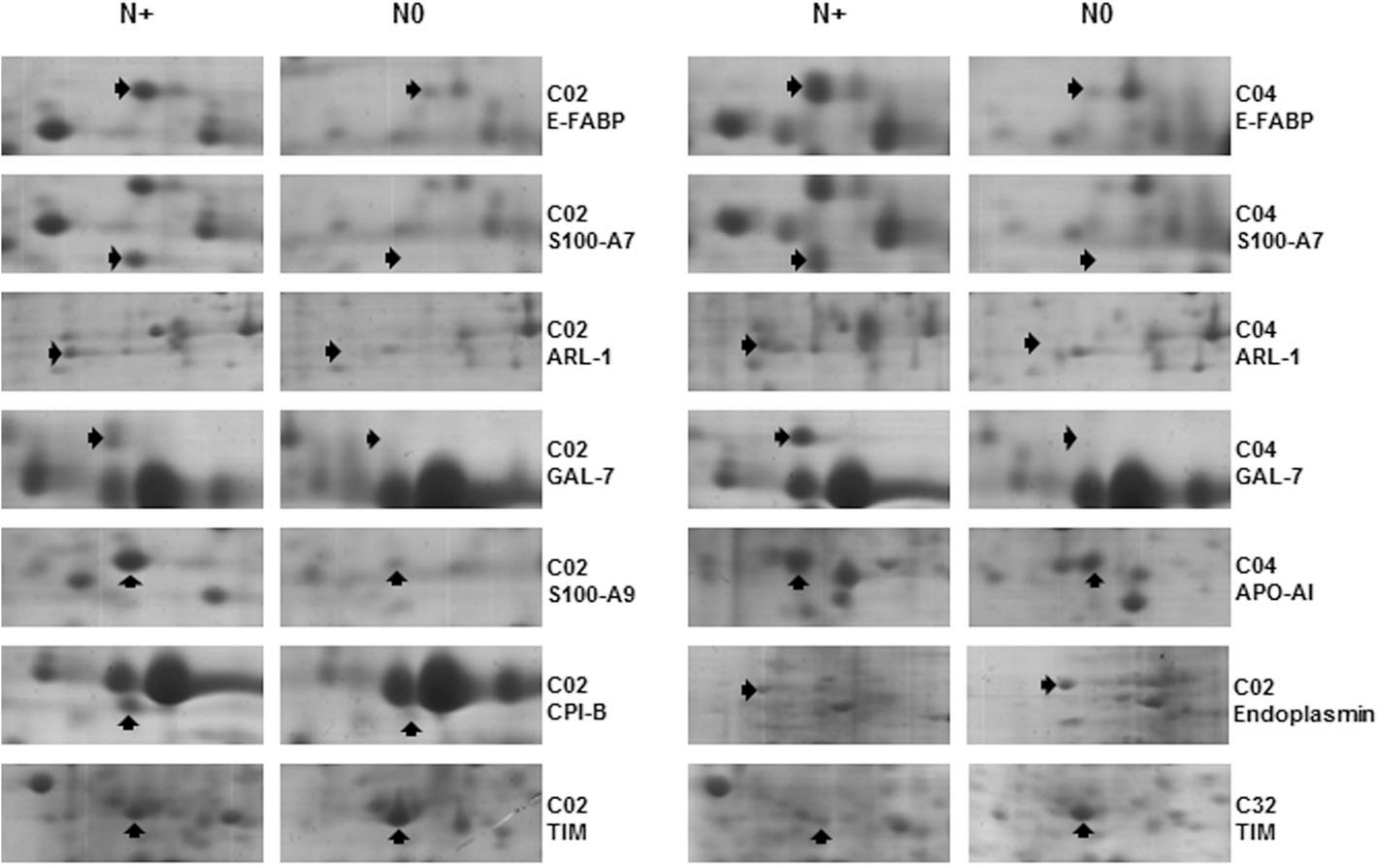
Partial 2-DE gel images of differentially expressed proteins in metastatic (N+) and non-metastatic (N0) lymph nodes of HNSCC. Anatomical subsites - C02 (tongue), floor of the mouth (C04) and larynx carcinomas (C32) - and protein symbols/names are provided to the right of each panel. Over and underexpressed proteins are indicated with arrows

The 1-DE data validated by Scaffold software allowed the identification of 39 differentially expressed proteins (≥ 2.0-fold change) between N0 and N+ lymph nodes, with over 99% confidence (as per the Scaffold algorithm, at least one unique peptide per protein) (Table 1). Using these parameters, the false discovery rate (FDR) for protein identification was 0.2%. The 2-DE analysis revealed 22 differentially expressed proteins between metastatic and non-metastatic lymph nodes (Student’s t test *p* < 0.05). Fourteen proteins were overexpressed and eight underexpressed in metastatic samples compared with non-metastatic ones (Table 2, Fig. 1, Additional file 3). Nine differentially expressed proteins were detected by both 1-DE and 2-DE (Apo-AI, CA-I, GSTP1–1, HspB1, hemoglobin subunit delta, CK1, profilin-1, TIM, protein S100-A9), four of them (Apo-AI, GSTP1–1, HspB1, CK1) overexpressed by 2-DE and underexpressed by 1-DE (Tables 1 and 2). Therefore, a total of 52 differentially expressed proteins were identified. Some of them exhibited a diverse 2-DE profile in metastasis of tongue, floor of the mouth and larynx carcinomas. For example, Apo-AI only showed differential expression in N+ lymph nodes of floor of the mouth tumors, and calreticulin and PDI in N+ lymph nodes of larynx carcinomas. Differences between C02 and C04, which are derived from the same anatomical subsite (oral cavity), were also observed, such as hemoglobin subunit delta, endoplasmin, LAP-3, Apo-AI, Ig gamma and kappa chains, and CK1 (Table 2).

**Table 1:** Under and overexpressed proteins identified by one-dimensional gel electrophoresis (1-DE) in metastatic (N+) and non-metastatic (N0) lymph nodes from laryngeal SCC patients. Proteins were separated by one-dimensional gel electrophoresis and identified by Q-TOF MS and Scaffold software according to quantitative value. Thirty-nine proteins with a fold change of at least 2.0 were considered with differential abundance between the categories

Protein name	Gene symbol	UniProt accession	Quantitative value		Fold change	N+
			N+	N0	N+/N0	
Actin, cytoplasmic 1	*ACTB*	P60709	1	8	0.1	Down
Serum albumin	*ALB*	P02768	2	14	0.1	Down
Apolipoprotein A-I	*APOA1*	P02647	0	10	< 2.0	Down
Rho GDP-dissociation inhibitor 1	*ARHGDIA*	P52565	0	2	< 2.0	Down
Rho GDP-dissociation inhibitor 2	*ARHGDIB*	P52566	0	4	< 2.0	Down
Flavin reductase (NADPH)	*BLVRB*	P30043	0	3	< 2.0	Down
Carbonic anhydrase 1	*CA1*	P00915	0	2	< 2.0	Down
Coactosin-like protein	*COTL1*	Q14019	0	2	< 2.0	Down
Glyceraldehyde-3-phosphate dehydrogenase	*GAPDH*	P04406	1	2	0.5	Down
Glutathione S-transferase P	*GSTP1*	P09211	0	2	< 2.0	Down
Hemoglobin subunit alpha	*HBA1/HBA2*	P69905	0	28	< 2.0	Down
Hemoglobin subunit beta	*HBB*	P68871	0	10	< 2.0	Down
Hemoglobin subunit delta	*HBD*	P02042	0	17	< 2.0	Down
Histone H2A type 1-A	*HIST1H2AA*	Q96QV6	0	2	< 2.0	Down
Histone H2B type 1-C/E/F/G/I	*HIST1H2BG*	P62807	0	3	< 2.0	Down
Histone H3.1	*HIST1H3A*	P68431	0	4	< 2.0	Down
Heat shock protein beta-1	*HSPB1*	P04792	0	3	< 2.0	Down
Keratin, type II cytoskeletal 1	*KRT1*	P04264	0	3	< 2.0	Down
Keratin, type I cytoskeletal 9	*KRT9*	P35527	0	2	< 2.0	Down
Myosin light polypeptide 6	*MYL6*	P60660	0	2	< 2.0	Down
Protein deglycase DJ-1	*PARK7*	Q99497	0	3	< 2.0	Down
Profilin-1	*PFN1*	P07737	0	5	< 2.0	Down
Peptidyl-prolyl cis-trans isomerase A	*PPIA*	P62937	1	6	0.2	Down
Peroxiredoxin-1	*PRDX1*	Q06830	1	6	0.2	Down
Peroxiredoxin-2	*PRDX2*	P32119	0	5	< 2.0	Down
Peroxiredoxin-6	*PRDX6*	P30041	0	2	< 2.0	Down
Proteasome subunit beta type-5	*PSMB5*	P28074	0	2	< 2.0	Down
Ras-related protein Rab-10	*RAB10*	P61026	1	2	0.5	Down
Ras-related protein Rab-5B	*RAB5B*	P61020	0	2	< 2.0	Down
Ras-related protein Rap-1A	*RAP1A*	P62834	0	2	< 2.0	Down
Transgelin-2	*TAGLN2*	P37802	0	2	< 2.0	Down
Triosephosphate isomerase	*TPI1*	P60174	2	11	0.2	Down
14–3-3 protein beta/alpha	*YWHAB*	P31946	0	7	< 2.0	Down
14–3-3 protein zeta/delta	*YWHAZ*	P63104	3	10	0.3	Down
Annexin A1	*ANXA1*	P04083	4	0	> 2.0	Up
Keratin, type I cytoskeletal 13	*KRT13*	P13646	3	0	> 2.0	Up
Keratin, type II cytoskeletal 6A	*KRT6A*	P02538	4	0	> 2.0	Up
Periostin	*POSTN*	Q15063	2	0	> 2.0	Up
Protein S100-A9	*S100A9*	P06702	10	0	> 2.0	Up

**Table 2:** Under and overexpressed proteins identified by two-dimensional gel electrophoresis (2-DE) in metastatic (N+) and non-metastatic (N0) lymph nodes from oral cavity SCC patients. Proteins were separated by two-dimensional electrophoresis and identified by MALDI-Q-TOF MS/MS. Twenty-two proteins were considered with differential abundance between the categories (Student’s t test *p* < 0.05). C02, C04, C32 columns correspond to pools from tongue (pools A and B), floor of the mouth (pools C and D) and larynx carcinomas (pools E and F), respectively. N+/N0: abundance ratio

Protein name	Gene symbol	UniProt accession	pI	Mass	Sequence coverage (%)	Score	Queries matched	Area^*a*^	C02	C04	C32	N+
									N+/N0			
Carbonic anhydrase 1 or CA-I	*CA1*	P00915	6.65	28,620	16	116	3	VII	0.4402	0.2033	0.2971	Down
Calreticulin or CRP55	*CALR*	P27797	4.29	48,283	9	167	3	IX			0.6211	Down
Hemoglobin subunit delta	*HBD*	P02042	7.97	16,028	19	64	2	IV		0.3906	0.1063	Down
Heat shock protein 90 kDa beta member 1 or endoplasmin	*HSP90B1*	P14625	4.76	92,696	4	69	3	I	0.4010		0.5364	Down
Cytosol aminopeptidase or LAP-3	*LAP3*	P28838	6.29	53,006	4	71	3	IX	0.4514		0.6685	Down
Protein disulfide-isomerase or PDI	*P4HB*	P07237	4.76	57,480	8	201	4	IX			0.6616	Down
Profilin-1	*PFN1*	P07737	8.48	15,085	10	50	2	IV	0.3568	0.4595	0.6419	Down
Triosephosphate isomerase or TIM	*TPI1*	P60174	6.51	26,807	8	51	1	VI	0.5020	0.3262	0.3373	Down
Aldo-keto reductase family 1 member B10 or ARL-1	*AKR1B10*	O60218	7.12	36,226	12	149	3	II	3.7339	2.2564		Up
Apolipoprotein A-I or Apo-AI	*APOA1*	P02647	5.56	30,759	11	104	3	VI		1.5333		Up
Cystatin-B or CPI-B	*CSTB*	P04080	7.90	11,224	24	64	2	XI	2.0971	1.9614		Up
Fatty acid-binding protein, epidermal or E-FABP	*FABP5*	Q01469	6.84	15,366	18	94	2	VIII	4.0016	7.4081	5.5954	Up
Glutathione S-transferase P or GSTP1–1	*GSTP1*	P09211	5.44	23,438	12	78	2	VI	2.3289	4.0385	2.9731	Up
Heat shock protein beta-1 or HspB1	*HSPB1*	P04792	5.98	22,826	20	105	3	III	3.2028	4.1256	4.5202	Up
Ig gamma-1 chain C region	*IGHG1*	P01857	8.46	36,596	9	71	2	X	4.1259		4.3046	Up
Ig kappa chain C region	*IGKC*	P01834	7.55	26,077	14	48	2	VII		2.5279	2.1303	Up
Keratin, type II cytoskeletal 1 or CK1	*KRT1*	P04264	8.16	66,018	4	82	2	VII		1.7525	2.0958	Up
Galectin-1 or Gal-1	*LGALS1*	P09382	5.34	14,917	8	54	1	V	1.8654	2.2229	1.4698	Up
Galectin-7 or Gal-7	*LGALS7B*	P47929	7.00	14,992	19	73	2	VIII	6.2867	2.5605	2.7244	Up
Protein S100-A7 or psoriasin	*S100A7*	P31151	6.26	11,433	34	103	3	VIII	6.4075	9.3486	5.3993	Up
Protein S100-A9 or calgranulin-B	*S100A9*	P06702	5.71	13,291	42	100	3	VIII	4.7500	5.9737	4.9505	Up
14–3-3 protein sigma or stratifin	*SFN*	P31947	4.64	27,874	15	98	3	III	13.4829	7.3989	27.6468	Up

^a^Areas are numbered as in Additional file 1

#### Metabolic pathways, associated ontologies and expression data

Clustering the set of 52 differentially expressed genes using DAVID, 16 annotation clusters were obtained, 6 of them with Bonferroni and Benjamini–Hochberg corrected *p*-values< 0.05 (Additional file 4). These clusters were related to regulation of cell-cell adhesion, cellular oxidant detoxification, response to reactive oxygen species, and membrane organization. When analyzed individually by HNdb tools, over and underexpressed proteins showed activities expected for lymph nodes containing metastatic cells, many being associated with angiogenesis, apoptosis, cell growth, cell migration, and development processes (Tables 3 and 4).

**Table 3:** Information on biological processes based on Gene ontology. Up-regulated proteins identified by proteomic analysis of positive lymph node samples. Proteins are referenced by their HGNC gene symbol

Biological process	Up-regulated proteins^*a*^
Angiogenesis	HSPB1
Apoptosis	LGALS1, S100A9, SFN
Anti-apoptosis	ANXA1, GSTP1, HSPB1
Autophagy	S100A9
Cell adhesion	
Cell-cell adhesion	APOA1, POSTN
Cell communication	
Signaling	ANXA1, APOA1, HSPB1, IGHG1, IGKC, LGALS1, S100A7, S100A9, SFN
Cell-cell signaling	S100A9
Cell growth	
Positive regulation of cell growth	S100A9
Cell migration or movement	
Cell motility	ANXA1, HSPB1, S100A9
Cytoskeleton organization	KRT13, S100A9
Developmental process	
System development	GSTP1, POSTN
Cell differentiation	ANXA1
Epidermis development	FABP5, S100A7, SFN
Metabolic process	AKR1B10, APOA1, FABP5, GSTP1
Protein metabolic process	APOA1, CSTB
Lipid metabolic process	APOA1, FABP5
Protein modification process	GSTP1, SFN
Response to stimulus	ANXA1, HSPB1
Defense response	KRT6A, S100A7, S100A9
Inflammatory response	ANXA1, APOA1, S100A9
Immune response	APOA1, IGHG1, IGKC
Response to ROS	GSTP1, S100A7
Transcription	S100A9, SFN
Translation	HSPB1
Transport	APOA1

^a^Name of up-regulated proteins:*AKR1B10* Aldo-keto reductase family 1 member B10, *ANXA1* Annexin A1, *APOA1* Apolipoprotein A-I, *CSTB* Cystatin-B, *FABP5* Fatty acid-binding protein, epidermal, *GSTP1* Glutathione S-transferase P, *HSPB1* Heat shock protein beta-1, *IGHG1* Ig gamma-1 chain C region, *IGKC* Ig kappa chain C region, *KRT6A* Keratin, type II cytoskeletal 6A, *KRT13* Keratin, type I cytoskeletal 13, *LGALS1* Galectin-1, *POSTN* Periostin, *S100A7* Protein S100A7, *S100A9* Protein S100A9, *SFN* 14–3-3 protein sigma

**Table 4:** Information on biological processes based on Gene ontology. Down-regulated proteins identified by proteomic analysis of positive lymph node samples. Proteins are referenced by their HGNC gene symbol

Biological process	Down-regulated proteins^*a*^
Apoptosis	CALR, YWHAB
Regulation of apoptosis	HBA1/2, HBB, P4HB, PRDX2
Anti-apoptosis	ALB, ARHGDIA, HSP90B1, PARK7, PSMB5, YWHAZ
Cell adhesion	ARHGDIA, ARHGDIB
Cell cycle	
Arrest	CALR, PSMB5
Cell communication	
Signaling	ACTB, ARHGDIA, ARHGDIB, CALR, HIST1H3A, HSP90B1, MYL6, PARK7, PSMB5, RAP1A, YWHAB, YWHAZ
Cell migration or movement	ARHGDIA, ARHGDIB, PFN1, PPIA
Cell motility	ACTB
Cell proliferation	
Positive regulation	CALR
Cytoskeleton organization	ARHGDIB, PFN1
Developmental process	ARHGDIB, MYL6
System development	PRDX1
Cell differentiation	CALR, RAP1A, TAGLN2
Epidermis development	KRT9
Metabolic process	ALB, BLVRB, CA1, GAPDH, HBA1/2, HBB, P4HB, PARK7, PSMB5, RAP1A
Protein metabolic process	ACTB, CALR, P4HB, RAP1A
Protein modification process	ACTB, CALR, HBA1/2, HBB, HIST1H3A, HSP90B1, PARK7, PFN1, PPIA, PSMB5, YWHAB
Monosaccharide metabolic process	GAPDH, TPI1
Oxidation-reduction process	HBA1/2, HBB
Response to stimulus	HBA1/2, PARK7, RAP1A
Defense response	COTL1, HIST1H2BG, HIST1H3A
Immune response	ACTB, ARHGDIB, HIST1H2BG, HSP90B1, PSMB5, YWHAB
Response to stress	HSP90B1, P4HB
Response to oxidative stress	HBA1/2, HBB, PARK7, PRDX1, PRDX2, PRDX6
Replication	
DNA replication	CALR, HIST1H3A, PPIA
Senescence	CALR
Transcription	CALR, HIST1H3A, PARK7, PRDX1, PRDX2, PSMB5, YWHAB, YWHAZ
Translation	CALR, GAPDH
Transport	ALB, CA1, HBA1/2, HBB, HBD, HSP90B1, RAB10, RAB5B, RAP1A

^a^Name of down-regulated proteins: *ACTB* Actin, cytoplasmic 1, *ALB* Serum albumin, *ARHGDIA* Rho GDP-dissociation inhibitor 1, *ARHGDIB* Rho GDP-dissociation inhibitor 2, *BLVRB* Flavin reductase (NADPH), *CA1* Carbonic anhydrase 1, *CALR* Calreticulin, *COTL1* Coactosin-like protein, GAPDH Glyceraldehyde-3-phosphate dehydrogenase, *HBA1/2* Hemoglobin subunit alpha, *HBB* Hemoglobin subunit beta, *HBD* Hemoglobin subunit delta, *HIST1H2BG* Histone H2B type 1-C/E/F/G/I, *HIST1H3A* Histone H3.1, *HSP90B1* Heat shock protein 90 kDa beta member 1, *KRT9* Keratin, type I cytoskeletal 9, *MYL6* Myosin light polypeptide 6, *P4HB* Protein disulfide-isomerase, *PARK7* Protein deglycase DJ-1, *PFN1* Profilin-1, *PPIA* Peptidyl-prolyl cis-trans isomerase A, *PRDX1* Peroxiredoxin-1, *PRDX2* Peroxiredoxin-2, *PRDX6* Peroxiredoxin-6, *PSMB5* Proteasome subunit beta type-5, *RAB10* Ras-related protein Rab-10, *RAB5B* Ras-related protein Rab-5B, *RAP1A* Ras-related protein Rap-1A, *TAGLN2* Transgelin-2, *TPI1* Triosephosphate isomerase, *YWHAB* 14–3-3 protein beta/alpha, *YWHAZ* 14–3-3 protein zeta/delta

Positive scores for gene-to-HNSCC association determined by HNdb hypergeometric test were referred to 30/52 genes encoding these proteins (Additional file 5). Seven out of 52 genes (*GSTP1*, *HSP90B1*, *HSPB1*, *PFN1*, *RAP1A*, *SFN*, *YWHAZ*) were assigned to KEGG pathways in cancer, cell migration and cell cycle, and in signaling networks involved in proliferation, cellular motility, apoptosis, cell adhesion, angiogenesis and genetic integrity.

### Immunodetection

**Fig. 2:**
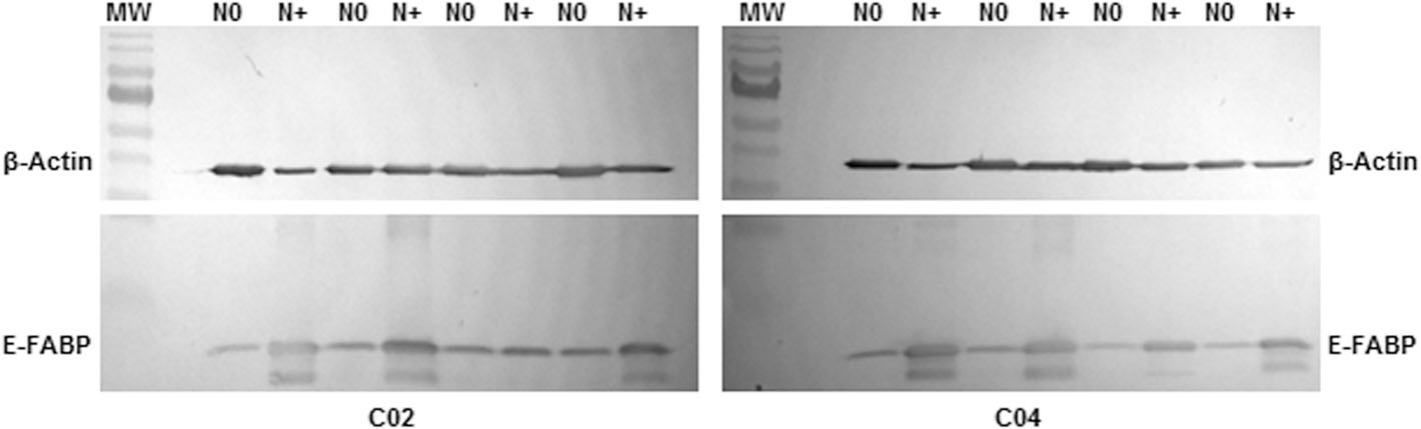
Immunodetection of E-FABP by Western blot. Representative Western blot illustrating the E-FABP expression in tumor-free (N0) and positive (N+) lymph nodes. β-actin was used as an internal control. MW=PageRuler Prestained Protein Ladder

**Fig. 3:**
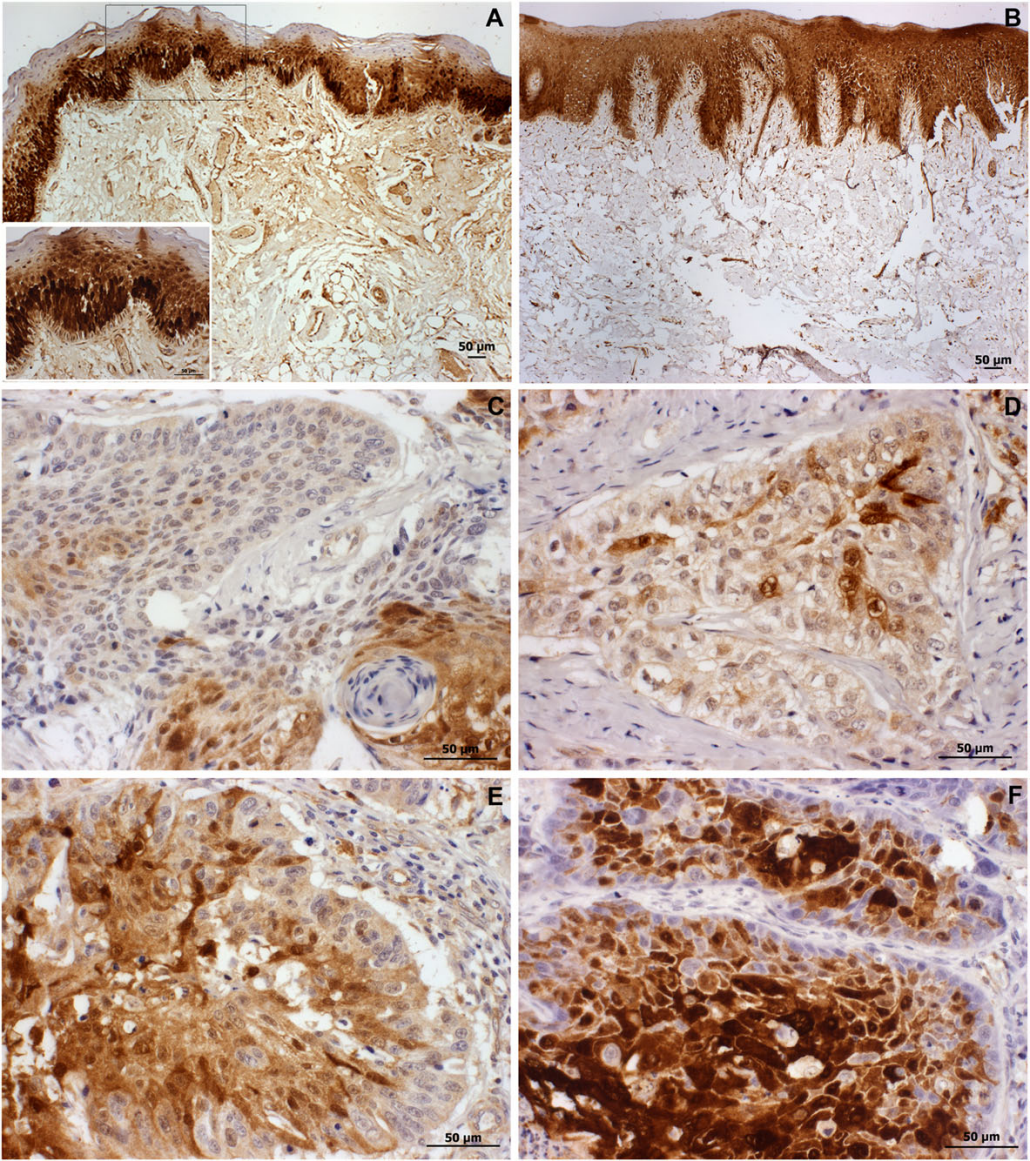
Immunohistochemical analysis of E-FABP expression in oral squamous cell carcinoma and non-tumoral (margin) samples. **a** Intense positivity of E-FABP in nucleus and cytoplasm of the basal and spinous layer of the normal epithelium, **b** reaching all epithelial layers. Immunolabeling intensity and proportion varied in tumor samples, with (**c**) expression in nests of well differentiated areas, **d** heterogeneous pattern with predominance of low intensity level in tumor cells; and also (**e**) moderate and (**f**) high intensity level of staining in nests. Scale bar indicates 50 μm

A protein related to invasive phenotype (E-FABP) and a potential cancer marker showing an unanticipated expression profile (annexin A1) were selected for validation by Western blotting and/or immunohistochemical assay. As expected, Western blot analysis revealed high levels of E-FABP in most N+ lymph nodes (10/11) when compared with N0 samples (0/11) (Fig. 2), which was confirmed by pixel density quantification using Image J software. In immunohistochemical assays, 41/65 primary tumor samples were considered positive for E-FABP (Fig. 3). Fisher’s exact test was used to estimate statistical difference between E-FABP positivity and clinicopathological parameters. There was no significant association between tumor size (*p* = 0.61), nodal metastasis (*p* = 0.80), pathologic TNM classification (*p* = 0.37), pathological grade (*p* = 0.20), lymphatic, and perineural invasion (*p* = 1.00; *p* = 0.36). Overall survival rate was compared with the expression of FABP5 using the Kaplan-Meier method, and the *P* value for the survival curve, determined by the log-rank test, was not statistically significant in the survival rates between positive and negative tumors (*p* = 0.88).

**Fig. 4:**
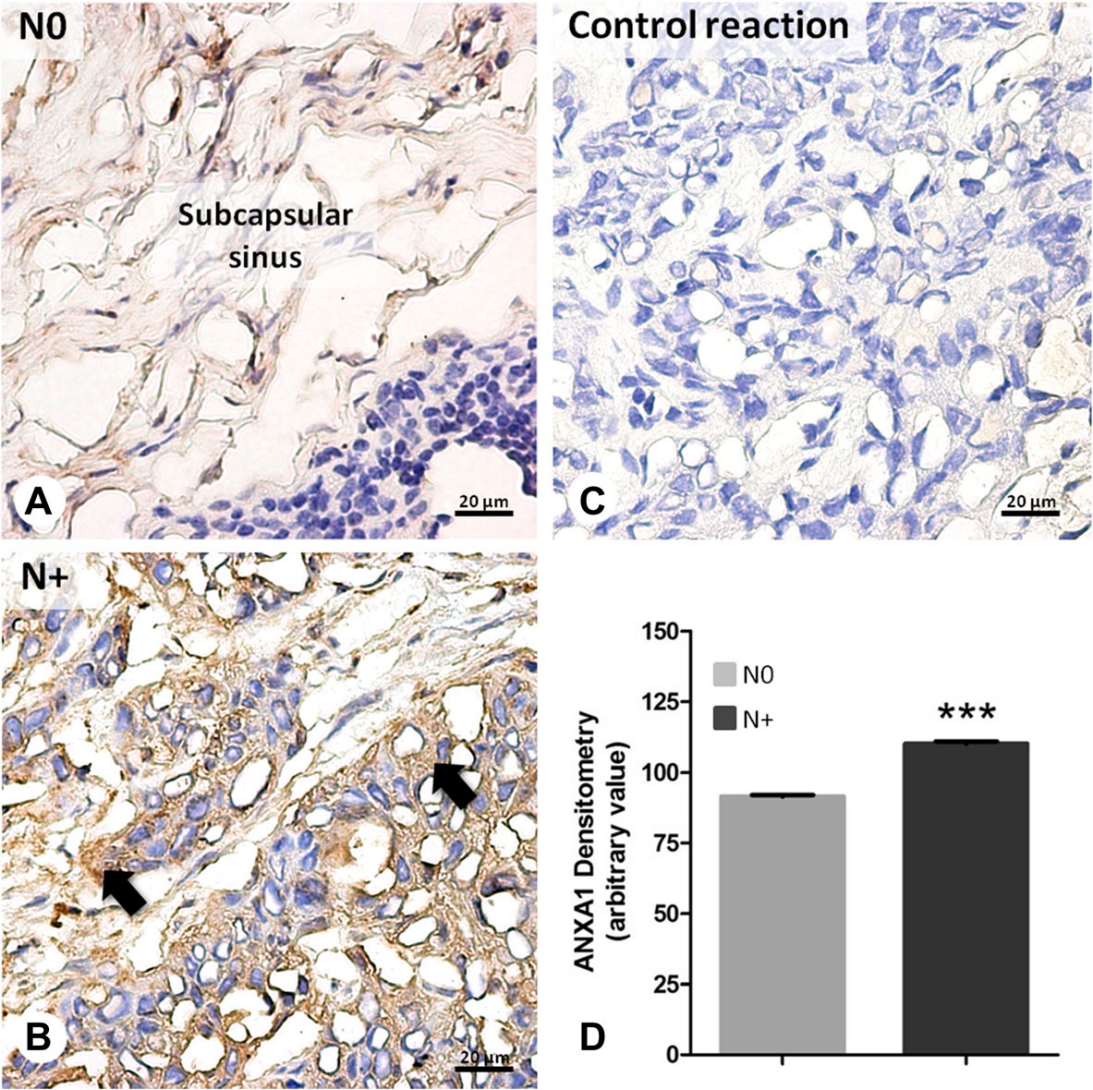
Immunohistochemical analysis of ANXA1 expression in lymph nodes from head and neck carcinomas. **a** Non-metastatic lymph node (N0) samples*:* constitutive expression of ANXA1 in the subcapsular sinus. **b** Metastatic lymph node (N+) samples*:* endogenous ANXA1 expression increased in the lymph node tissue and in the metastatic cells (arrows). **c** Negative control of reaction. Sections: 2 μm. Counterstain: Hematoxylin. **d** Densitometry of ANXA1. Values expressed as mean ± S.E.M. *** *p* < 0,001

Immunohistochemical analysis of ANXA1 was carried out on metastatic and non-metastatic lymph node samples. Constitutive ANXA1 expression was observed in the subcapsular sinus of N0 lymph nodes (Fig. 4a). In the N+ samples, its expression was increased, especially in the loose conjunctive tissue, which constitutes the subcapsular sinus, above the external cortex of the lymph node. In these metastatic samples, epithelial cells showed a more intense cytoplasmatic expression of ANXA1 compared to control biopsies (Fig. 4b). No immunostaining was detected in the negative control (Fig. 4c).

ANXA1 was not validated by Western blot. Due to the limited amount of protein from lymph node samples, the detection by immunohistochemistry was prioritized, since it is a more sensitive and specific assay and can be performed on paraffin-embedded sections.

## Discussion

In the present study, we investigated the proteomic profile of lymph node metastasis from squamous cell carcinomas of tongue, floor of the mouth and larynx, by using one- and two-dimensional electrophoresis and mass spectrometry analysis. Fifty-two proteins were differentially expressed in metastatic compared with non-metastatic lymph nodes analyzed by 1-DE and 2-DE. Western blot and/or immunohistochemical analysis confirmed the results for two representative proteins (E-FABP and annexin A1). Although the performance of 1-DE was better than that of 2-DE, several recent studies using 2-DE technique present consistent results [36–40] and show that it is still an important top-down analytical approach [41]. Anyhow, 1-DE is also unable to completely resolve complex mixtures of proteins.

Some of the over and underexpressed proteins may play an important role in the head and neck tumorigenesis and metastatization processes. For example, aldo-keto reductase ARL-1 is a potential biomarker for non-small cell lung cancer of smokers [42] and, therefore, may be involved in the pathogenesis of tobacco-related cancers [43], including HNSCCs. Other proteins have already been associated with HNSCC by several authors [44–46] and by our group, particularly annexin A1 [47, 48], fatty acid-binding protein E-FABP [49, 50], heat shock protein beta-1 [51, 52], galectin-1 [53, 54], glutathione S-transferase P [55, 56], keratin, type I cytoskeletal 13 [57, 58], peptidyl-prolyl cis-trans isomerase A [59, 60], periostin [61], protein deglycase DJ-1 [62, 63], protein S100A7 [64, 65] and Ras-related protein RAP-1A [66]. In fact, the genes encoding some of these proteins showed the highest scores for gene-to-HNSCC association determined by HNdb hypergeometric test, namely *FABP5*, *S100A7*, *ANXA1*, *LGALS1*, *PARK7*, *GSTP1*, with scores ranging from 1.88 to 83. The expression pattern of these proteins was also evaluated using The Human Protein Atlas (https://www.proteinatlas.org), a public database containing protein expression information based on approximately 700 antibodies combined with transcriptomics data from The Cancer Genome Atlas - TCGA (average fragments per kilobase of transcript per million mapped reads - FPKM) [67]. Because Protein Atlas has very few samples analyzed by immunohistochemistry and joins different subsites of head and neck carcinomas, the data showed low concordance with our findings. Otherwise, our findings showed higher concordance with the average FPKM values, especially for upregulated proteins. Similarly to what our group and other authors (44, 45, 81) observed for ANXA1/annexin A1 in primary tumors, Protein Atlas and TCGA refer a low expression of this protein in primary HNSCC.

Some differences were observed between lymph node samples from dissimilar anatomic subsites, which support a molecular heterogeneity for HNSCC metastasis, also previously reported by us for the primary tumors [68]. The differences in expression of, for example, APO-AI, calreticulin, CK1, endoplasmin, LAP-3, PDI, may affect tumor progression and drug response because these proteins are involved in signaling, cell proliferation, response to hypoxia and oxidative stress.

In regard to the metastasis environment, many questions remain. What proteins are predictive biomarkers for regional metastasis in HNSCC? What features were previously selected and expressed in cells leaving the primary tumor? After arriving in the lymph nodes, what would be the new challenge for tumor cells? Tentative answers to these questions may be exemplified by the findings we obtained for galectin-1 and psoriasin. These proteins have been shown to be associated with hypoxia [69–71], a common adverse condition faced by metastatic, as well as primary tumor cells. The findings of Chaudary and Hill [72] reinforce the idea that ‘hypoxia-related’ factors regulate lymph node metastasis under intermittent hypoxic conditions. According to these authors, lymphatic vessels occur more often only in the periphery of tumors; these regions of acute hypoxia may stimulate the cells to spread through lymphatic vessels, leading to increased lymph node metastasis [73].

Concerning epidermal-type fatty acid-binding protein (E-FABP), our proteomic approach detected that this member of the fatty acid-binding protein family is over-expressed in lymph node metastasis, a result supported by Western blotting experiments in tumor samples. These findings are somewhat in disagreement with those of Uma and collaborators [49], who reported a down-regulation of *FABP5* in metastatic lymph nodes compared to the corresponding primary tumors. These discordant results can be explained by the fact that Uma’s group analyzed transcripts while the present work evaluated gene expression at the protein level. General correlations between the levels of RNA and the corresponding proteins have been observed, but even with stringent methods partial or reverse correlations are also detected, probably due to regulatory mechanisms or variable accuracy on the RNA level, as reviewed by Gry and collaborators [74].

However, similarly to our findings, *FABP5*/E-FABP overexpression in HNSCC lymph node metastasis has been observed by others [50, 75]. Increased serum reactivity to E-FABP in HNSCC patients [75], and association of higher E-FABP levels with HPV-positive oral and oropharyngeal carcinomas [76] and with cell proliferation and invasiveness [50] have also been found. In respect to HPV status, our group have previously studied a cohort of more than 1000 HNSCC cases to determine the serological response to oncoproteins of HPV16 and 400 HNSCC cases to investigate HPV16 DNA in tumor samples. The results showed a low prevalence of HPV16 DNA and HPV16 E6 and E6/E7 antibodies in oral and larynx carcinomas [77]. Given that the patients analyzed by the present study represent a subset included in this previous report, we can hypothesize that *FABP5*/E-FABP findings are not related to the HPV status in our cohorts. Regarding other neoplasms, a high expression of E-FABP was detected in tumor tissues, serum [78] and urinary extracellular vesicles from patients with high prostate cancer [79] as well as in cervical cancer tissues, and significantly correlated with lymph node metastasis, lymphovascular space invasion, stage and tumor size [80].

E-FABP is a cytosolic lipid binding protein of epidermal cells that uptakes, binds and transports long chain fatty acids to cell organelles. The study of Bao et al. [81] demonstrated that E-FABP overexpression results in an increase in the levels of fatty acid uptake and transport into the nucleus, and also in tumor-promoting activity. The authors suggested that such tumorigenic activity is due to the activation of the nuclear receptor peroxisome proliferator-activated receptor gamma (PPARγ) by fatty acids resulting in upregulation of genes involved in angiogenesis, apoptosis suppression and invasion.

E-FABP and protein S100-A7, both over-expressed in our N+ samples, stabilize the level of each other, and colocalize in focal adhesion-like structures in response to calcium, possibly as part of a protein complex with an important role in the metastatic process [82]. Abnormal expression of S100 proteins has already been detected in metastasis of colorectal cancers [83] and associated with lymph node positive tumors and invasive/migratory phenotype [84]. Similar results have been observed for apolipoprotein A-I, which also shows high expression in lymph node metastasis of primary colonic adenocarcinomas [85] and saliva and serum from HNSCC [86].

Some proteins identified by us presented opposite results to those of the literature regarding their expression in cancer cells. For example, endoplasmin and triosephosphate isomerase were found downregulated in our metastatic lymph node samples, and upregulated in tumor samples analyzed by Nomura H et al. [87] and Polachini GM et al. [88], which may be explained by the effect of the lymph node immune environment in modulating tumor growth or in metabolic reprogramming of the metastatic cell considering the blood flow, oxygen and nutrient supplies in the secondary site.

Annexin A1, a member of the annexin superfamily, has been observed underexpressed in primary HNSCC studied by us [47] and by others [48, 88]. However, our mass spectrometry and immunohistochemical analyses showed that it is overexpressed in positive lymph nodes. This is the first direct evidence of annexin A1 overexpression in lymph node metastasis of head and neck cancer. Annexin A1 is a protein involved in inflammation [89], apoptosis [90], cell differentiation [91], migration, invasion [92], and signaling [93]. It is a substrate of growth factors and kinases and exhibits abnormal (high or low) levels in several tumors and inflammatory conditions (reviewd by [94–96]. In HNSCC, ANXA1 down-regulation has been associated with poor differentiation and advanced stages [91, 97], but also with early stages, at least in laryngeal tumorigenesis [47]. In breast cancer, ANXA1 is highly expressed and modulates activation of M2 macrophage, which in its turn promotes angiogenesis, tumor progression and adaptive immune response [98]. Increased levels of annexin A1 are also observed in bronchoalveolar lavage fluid and correlated with lymphatic invasion and malignant progression of lung cancer [99]. A similar expression pattern has been described in hypoxic conditions, when it binds to formyl peptide receptors and induces cell invasion [100]. Thus, the conflicting data between our lymph node samples and previously analyzed primary tumors may indicate a complex annexin A1-cancer relationship, with distinct actions depending on the cell type, as well discussed by Tu Y et al. [101].

At the present time, there are few biomarkers that can predict progression of head and neck carcinomas. Although the lymph node status is still the most important predictor, occult micrometastases may not be detected by the routine histopathological examination of neck dissection specimens. Therefore, markers of a well characterized metastatic phenotype could help to identify reduced numbers of neoplastic cells in lymph nodes or even before homing to and colonizing lymph nodes – the circulating tumor cells (CTCs) - using non-invasive tests. However, the number of CTCs in HNSCC patients is low and enrichment strategies need to be performed to increase CTC concentration and, consequently, to facilitate their detection and characterization [102]. Recently, Kulasinghe and collaborators [103, 104] demonstrate that CTC clusters may actually be an important HNSCC prognostic marker. In vivo and in vitro experiments should validate this finding and probably will help to clarify why lymphatic vessels and regional lymph nodes are the preferential target sites of head and neck carcinoma cells.

## Conclusions

The present study has some methodological limitations. First, the number of expressed proteins in complex biological samples is many orders of magnitude greater than the total number of spots visible in 2-DE gels after staining. Therefore, only a small percentage of the total sample proteome is available for comparisons. Second, the usage of pools may miss relevant differences between samples. Third, the methodology of the present study does not allow to determine whether differences between groups are cause or consequence of tumorigenesis, an issue that should be the aim of future analyses, such as the analysis of cells before lymph node colonization - the circulating tumor cells.

Despite methodological limitations**,** this study provides, for the first time, direct evidence of annexin A1 overexpression in lymph node metastasis of head and neck cancer and adds information that may be useful for diagnosing metastatic disease. The results on the expression of heat shock proteins and enzymes of the glycolytic pathway suggest an effect of the lymph node environment in controlling tumor growth or in metabolic reprogramming of the metastatic cell. In addition, the observation of several proteins with differential expression between lymph node metastasis from tongue, floor of the mouth and larynx carcinomas reinforces the idea that head and neck sites and subsites are dissimilar entities whose behavior may be influenced by micro-environmental factors including the lymphatic network. Taken together, the results from this study contributed to our understanding of the metastatic phenotype of HNSCC and provided novel potential targets for diagnostic in metastatic head and neck squamous cell carcinomas.

## Additional files

Additional file 1: Clinicopathological features of 105 HNSCC patients. (DOC 165 kb)

Additional file 2: Pools organized into groups according to anatomical site and presence (N+) or absence (N0) of tumor cells in lymph node. (DOC 39 kb)

Additional file 3: Two-dimensional electrophoresis maps of human lymph node proteins from HNSCC patients. (TIF 11816 kb)

Additional file 4: Enriched categories for 52 genes mapped to 53 DAVID identifiers. (XLSX 51 kb)

Additional file 5: Positive scores for gene-to-HNSCC association determined by HNdb hypergeometric test. (XLS 33 kb)

## Abbreviations

1-DE: One-Dimensional Gel Electrophoresis
2-DE: Two-Dimensional Gel Electrophoresis
ACN: Acetonitrile
ANXA1: Annexin A1
CHAPS: 3-[(3-Cholamidopropyl) Dimethylammonio]-1-Propanesulfonate Hydrate
DAB: 3,3′-Diaminobenzidine
DTT: Dithiothreitol
E-FABP: Fatty Acid-Binding Protein, Epidermal
EMT: Epithelial-Mesenchymal Transition
FDR: False Discovery Rate
FFPE: Formalin-Fixed, Paraffin-Embedded Tissue
HCL: Hydrochloric Acid
HGNC: Hugo Gene Nomenclature Committee
HNSCC: Head and Neck Squamous Cell Carcinoma
KEGG: Kyoto Encyclopedia of Genes and Genomes
MALDI: Matrix Assisted Laser Desorption Ionization
MS: Mass Spectrometry
N +: Lymph Node positive for neoplastic cells.
N0: Lymph Node negative for neoplastic cells
PBS: Phosphate-Buffered Saline
PPARγ: Peroxisome Proliferator-Activated Receptor Gamma
PVDF: Polyvinylidene Difluoride
Q-TOF: Quadrupole Ion Trap - Time Of Flight
SDS: Sodium Dodecyl Sulfate
TFA: Trifluoroacetic Acid
TMA: Tissue Microarray
TNM: Tumor-Node-Metastases
