# Supplementary material for: Differentially expressed proteins in positive versus negative HNSCC lymph nodes
Source: BMC Med Genomics. 2018 Aug 29;11:73. doi: 10.1186/s12920-018-0382-6 (PMC6114741; doi:10.1186/s12920-018-0382-6)
Supplement: Supplementary file 1 — Clinicopathological features of 105 HNSCC patients. (DOC 165 kb) [file 12920_2018_382_MOESM1_ESM.doc]

**Additional file 1.** **Clinicopathological features of 105 HNSCC patients.**

| **Case** | **Age (yrs)** | **Smoking status** | **Alcohol consumption** | **Site**  **ICD-10***^a^* | **Pathological TNM** | **Technique** |
| --- | --- | --- | --- | --- | --- | --- |
| CP1/0066 | 72 | current smoker | current drinker | C32 | T4N2BM0 | 1-DE/2-DE |
| CP3/0290 | 50 | former smoker | former drinker | C32 | T4N2cM0 | 1-DE/2-DE |
| CP1/0042 | 70 | current smoker | current drinker | C32 | T4N2CM0 | 1-DE/2-DE/IH |
| CP3/0041 | 52 | current smoker | never | C32 | T4N2cM0 | 1-DE/2-DE/WB |
| CP3/0105 | 67 | former smoker | current drinker | C32 | T4N2cM0 | 1-DE/2-DE/WB |
| CP1/0058 | 50 | current smoker | former drinker | C32 | T4N2CM0 | 1-DE/2-DE/WB/IH |
| CP1/0175 | 61 | current smoker | current drinker | C32 | T4N0M0 | 1-DE/2-DE/WB/IH |
| CP1/0180 | 74 | former smoker | former drinker | C32 | T4N0M0 | 1-DE/2-DE/WB/IH |
| CP3/0301 | 54 | current smoker | current drinker | C32 | T4aN0M0 | 1-DE/2-DE/WB/IH |
| CP1/0095 | 63 | current smoker | current drinker | C04 | T3N2CM0 | 2-DE |
| CP1/0225 | 48 | current smoker | current drinker | C04 | T3N2CM0 | 2-DE |
| CP1/0230 | 63 | current smoker | current drinker | C04 | T4N2CM0 | 2-DE |
| CP1/0240 | 75 | current smoker | current drinker | C04 | T2N0M0 | 2-DE |
| CP3/0113 | 67 | never | current drinker | C02 | T4N1M0 | 2-DE |
| CP1/0154 | 59 | current smoker | former drinker | C04 | T4N2bM0 | 2-DE |
| CP1/0280 | 45 | current smoker | current drinker | C02 | T3N2BM0 | 2-DE/IH |
| CP1/0057 | 57 | current smoker | former drinker | C02 | T4N2BM0 | 2-DE/WB |
| CP1/0086 | 61 | current smoker | current drinker | C04 | T1N0M0 | 2-DE/WB |
| CP1/0151 | 47 | current smoker | current drinker | C02 | T3N0M0 | 2-DE/WB |
| CP1/0183 | 72 | current smoker | current drinker | C04 | T4N2BM0 | 2-DE/WB |
| CP1/0248 | 50 | current smoker | current drinker | C04 | T4N0M0 | 2-DE/WB |
| CP1/0273 | 56 | never | never | C02 | T4N2BM0 | 2-DE/WB |
| CP1/0281 | 57 | current smoker | current drinker | C02 | T4N2BM0 | 2-DE/WB |
| CP1/0017 | 55 | current smoker | former drinker | C02 | T2N0M0 | 2-DE/WB/IH |
| CP1/0053 | 52 | former smoker | former drinker | C04 | T2N0M0 | 2-DE/WB/IH |
| CP1/0055 | 56 | current smoker | current drinker | C04 | T4N2BM0 | 2-DE/WB/IH |
| CP1/0070 | 48 | current smoker | current drinker | C04 | T4N1M0 | 2-DE/WB/IH |
| CP1/0171 | 70 | never | current drinker | C04 | T2N1M0 | 2-DE/WB/IH |
| CP1/0212 | 69 | current smoker | current drinker | C02 | T4N0M0 | 2-DE/WB/IH |
| CP1/0232 | 79 | never | never | C02 | T3N0M0 | 2-DE/WB/IH |
| CP3/0332 | 56 | current smoker | current drinker | C02 | T3N2bM0 | 2-DE/WB/IH |
| CP3/0094 | 68 | current smoker | former drinker | C04 | T4N0M0 | 2-DE/WB/IH |
| CP3/0083 | 55 | current smoker | current drinker | C02 | T1N1M0 | IH |
| CP3/0087 | 52 | current smoker | current drinker | C02 | T2N2cM0 | IH |
| CP3/0139 | 50 | current smoker | current drinker | C02 | T2N2aM0 | IH |
| CP3/0293 | 58 | current smoker | current drinker | C02 | T3N0M0 | IH |
| CP3/0371 | 42 | current smoker | current drinker | C04 | T4aN0M0 | IH |
| CP3/0452 | 47 | current smoker | never | C03 | T4aN0M0 | IH |
| CP3/0484 | 49 | current smoker | former drinker | C02 | TxN2bM0 | IH |
| CP3/0486 | 56 | current smoker | current drinker | C02 | T3N0M0 | IH |
| CP2/0003 | 79 | current smoker | never | C03 | T2N1M0 | TMA |
| CP2/0008 | 54 | current smoker | current drinker | C02 | T2N0M0 | TMA |
| CP2/0010 | 55 | current smoker | current drinker | C06 | T3N0M0 | TMA |
| CP2/0013 | 68 | current smoker | former drinker | C02 | T4N2cM0 | TMA |
| CP2/0019 | 63 | former smoker | current drinker | C04 | T3N2M0 | TMA |
| CP2/0023 | 54 | current smoker | former drinker | C02 | T3N0M0 | TMA |
| CP2/0029 | 70 | current smoker | current drinker | C04 | T2N0M0 | TMA |
| CP2/0036 | 55 | current smoker | current drinker | C06 | T4N2bM0 | TMA |
| CP2/0039 | 56 | current smoker | former drinker | C02 | T2N0M0 | TMA |
| CP2/0040 | 41 | never | never | C02 | T1N2bM0 | TMA |
| CP2/0071 | 49 | current smoker | current drinker | C04 | T4N2bM0 | TMA |
| CP2/0074 | 40 | current smoker | current drinker | C04 | T4N0M0 | TMA |
| CP2/0081 | 62 | former smoker | never | C03 | T4N0M0 | TMA |
| CP2/0087 | 69 | former smoker | current drinker | C03 | T4N2bM0 | TMA |
| CP2/0093 | 55 | current smoker | current drinker | C04 | T2N0M0 | TMA |
| CP2/0094 | 47 | current smoker | current drinker | C02 | T3N1M0 | TMA |
| CP2/0114 | 51 | former smoker | current drinker | C04 | T4N2M0 | TMA |
| CP2/0115 | 63 | former smoker | former drinker | C06 | T4N0M0 | TMA |
| CP2/0117 | 35 | current smoker | current drinker | C02 | T2N2bM0 | TMA |
| CP2/0120 | 41 | former drinker | never | C02 | T2N1M0 | TMA |
| CP2/0125 | 53 | current smoker | current drinker | C03 | T4N0M0 | TMA |
| CP2/0130 | 49 | current smoker | former drinker | C03 | T2N0M0 | TMA |
| CP2/0132 | 48 | current smoker | current drinker | C02 | T2N1M0 | TMA |
| CP2/0133 | 46 | current smoker | current drinker | C02 | T4N1M0 | TMA |
| CP2/0144 | 65 | former smoker | former drinker | C06 | T2N0M0 | TMA |
| CP2/0149 | 58 | current smoker | former drinker | C04 | T4N1M0 | TMA |
| CP2/0152 | 46 | current smoker | current drinker | C02 | T4N2bM0 | TMA |
| CP2/0166 | 62 | former smoker | never | C03 | T4N2bM0 | TMA |
| CP2/0168 | 70 | former smoker | never | C04 | T2N0M0 | TMA |
| CP2/0169 | 42 | current smoker | current drinker | C04 | T2N2bM0 | TMA |
| CP2/0170 | 34 | current smoker | current drinker | C04 | T4N2bM0 | TMA |
| CP2/0182 | 38 | former smoker | former drinker | C06 | T4N0M0 | TMA |
| CP2/0185 | 64 | former smoker | current drinker | C02 | T2N0M0 | TMA |
| CP2/0188 | 41 | current smoker | current drinker | C02 | T4N2bM0 | TMA |
| CP2/0195 | 47 | current smoker | current drinker | C04 | T1N1M0 | TMA |
| CP2/0196 | 69 | current smoker | former drinker | C04 | T4N2cM0 | TMA |
| CP2/1002 | 59 | current smoker | current drinker | C02 | T2N2CM0 | TMA |
| CP2/1003 | 59 | former smoker | never | C03 | T2N0M0 | TMA |
| CP2/1008 | 68 | former smoker | former drinker | C03 | T4N1M0 | TMA |
| CP2/1010 | 58 | current smoker | former drinker | C02 | T1N0M0 | TMA |
| CP2/1012 | 55 | current smoker | former drinker | C02 | T3N0M0 | TMA |
| CP2/1019 | 37 | current smoker | current drinker | C04 | T4N2CM0 | TMA |
| CP2/1021 | 67 | current smoker | current drinker | C03 | T4N1M0 | TMA |
| CP2/1022 | 44 | current smoker | current drinker | C02 | T3N0M0 | TMA |
| CP2/1030 | 61 | current smoker | current drinker | C02 | T2N0 | TMA |
| CP2/1032 | 57 | never | never | C03 | T4N0M0 | TMA |
| CP2/1036 | 57 | current smoker | current drinker | C02 | T4N1M0 | TMA |
| CP2/1041 | 42 | current smoker | current drinker | C04 | T4N2cM0 | TMA |
| CP2/1043 | 69 | current smoker | current drinker | C03 | T4N2bM0 | TMA |
| CP2/1044 | 51 | current smoker | former drinker | C02 | T2N2AM0 | TMA |
| CP2/1051 | 56 | current smoker | former drinker | C06 | T3N2bM0 | TMA |
| CP2/1065 | 59 | former smoker | current drinker | C02 | T2N0M0 | TMA |
| CP2/1069 | 46 | current smoker | former drinker | C02 | T2N0M0 | TMA |
| CP2/1071 | 44 | current smoker | current drinker | C02 | T4N2CM0 | TMA |
| CP2/1073 | 58 | current smoker | current drinker | C02 | T2N0M0 | TMA |
| CP2/1074 | 78 | former smoker | former drinker | C02 | T3N0M0 | TMA |
| CP2/1080 | 64 | current smoker | former drinker | C04 | T2N0M0 | TMA |
| CP2/1099 | 56 | current smoker | current drinker | C04 | T4N2bM0 | TMA |
| CP2/1104 | 67 | current smoker | current drinker | C02 | T3N2bM0 | TMA |
| CP2/1109 | 52 | former smoker | former drinker | C04 | T2N0M0 | TMA |
| CP2/1111 | 42 | current smoker | current drinker | C03 | T3N1M0 | TMA |
| CP2/1112 | 75 | never | never | C04 | T4N2bM0 | TMA |
| CP2/1113 | 53 | current smoker | never | C04 | T1N0M0 | TMA |
| CP2/1114 | 58 | current smoker | current drinker | C03 | T2N1M0 | TMA |
| CP2/1120 | 47 | current smoker | current drinker | C04 | T3N0M0 | TMA |

^a^Subsites of HNSCC according to WHO: C02= **tongue; C03= gum; C04=floor of mouth; C06=other and unspecified parts of mouth; C32=larynx.**

Abbreviations: 1-DE=one-dimensional electrophoresis; 2-DE=two-dimensional electrophoresis; WB=Western blot; IH=immunohistochemistry in a single paraffin block; TMA=immunohistochemistry on tissue microarray.
