# Supplementary material for: Differentially expressed proteins in positive versus negative HNSCC lymph nodes
Source: BMC Med Genomics. 2018 Aug 29;11:73. doi: 10.1186/s12920-018-0382-6 (PMC6114741; doi:10.1186/s12920-018-0382-6)
Supplement: Supplementary file 2 — Pools organized into groups according to anatomical site and presence (N+) or absence (N0) of tumor cells in lymph node. (DOC 39 kb) [file 12920_2018_382_MOESM2_ESM.doc]

**Additional file 2.** **Pools organized into** groups according **to anatomical site and presence (N+) or absence (N0) of tumor cells in lymph node.**

| **Pool** | **Site^a^** | **Case** | **Group** | **Technique** |
| --- | --- | --- | --- | --- |
| **A** | C02 | CP1/0017, CP1/0151, CP1/0212, CP1/0232 | N0 | 2DE |
| **B** | C02 | CP1/0057, CP1/0273, CP1/0280, CP1/0281, CP3/0113, CP3/0332 | N+ | 2-DE |
| **C** | C04 | CP1/0053, CP1/0086, CP1/0240, CP1/0248, CP3/0094 | N0 | 2-DE |
| **D** | C04 | CP1/0055, CP1/0070, CP1/0095, CP1/0154, CP1/0171, CP1/0183, CP1/0225, CP1/0230 | N+ | 2-DE |
| **E** | C32 | CP1/0175, CP1/0180, CP3/0301 | N0 | 1-DE/2-DE |
| **F** | C32 | CP1/0042, CP1/0058, CP1/0066, CP1/0041, CP3/0105, CP3/0290 | N+ | 1-DE/2-DE |

^a^Site of primary tumor: C02=**tongue; C04=floor of mouth; C32 =larynx**

Abbreviations: 1-DE=one-dimensional electrophoresis; 2-DE=two-dimensional electrophoresis.
