# Supplementary figures and images for: Differentially expressed proteins in positive versus negative HNSCC lymph nodes
Source: BMC Med Genomics. 2018 Aug 29;11:73. doi: 10.1186/s12920-018-0382-6 (PMC6114741; doi:10.1186/s12920-018-0382-6)

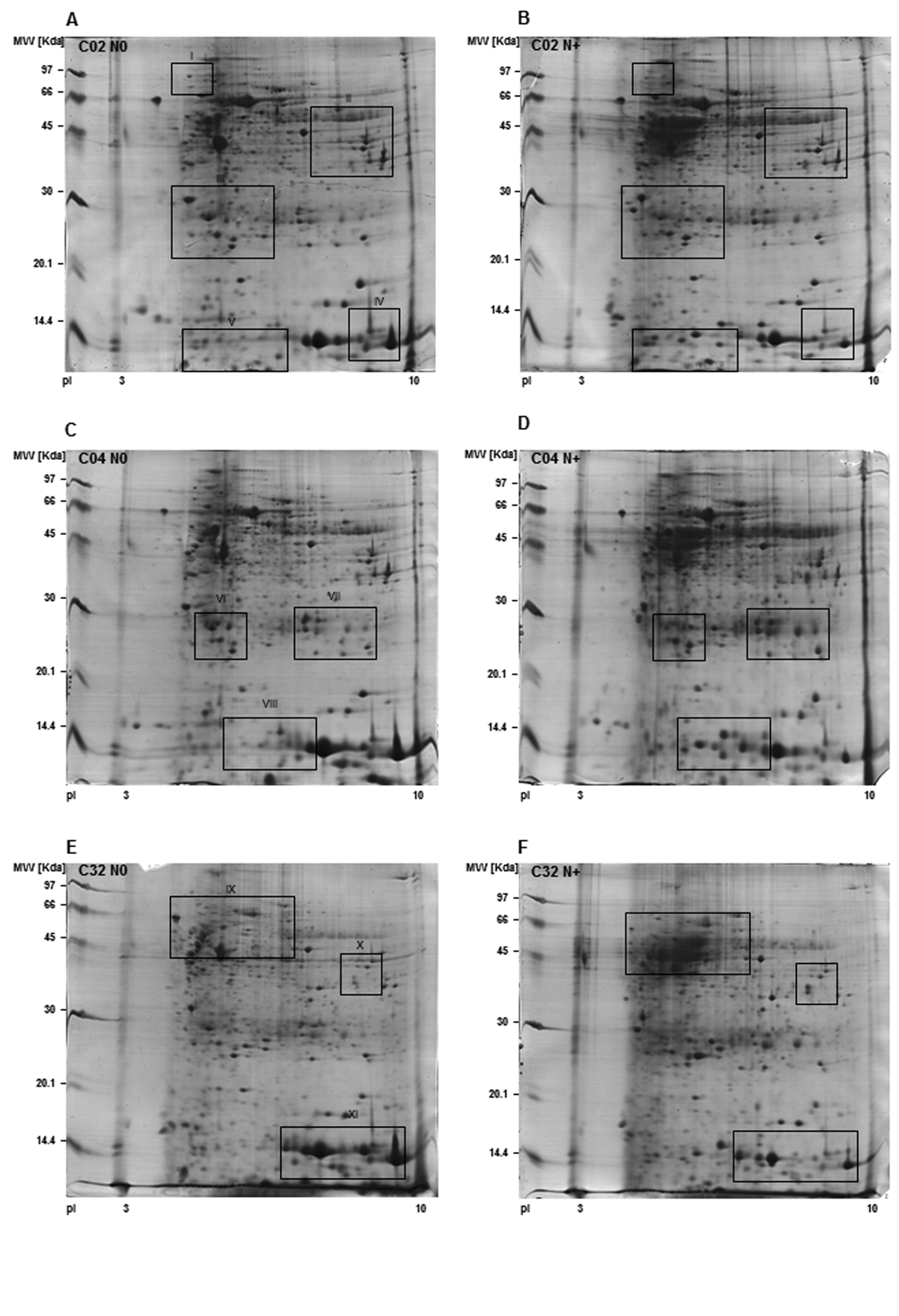

Supplement: Supplementary file 3 — Two-dimensional electrophoresis maps of human lymph node proteins from HNSCC patients. (TIF 11816 kb) [file 12920_2018_382_MOESM3_ESM.tif]
